# Association between blood urea nitrogen levels and the risk of diabetes mellitus in Chinese adults: secondary analysis based on a multicenter, retrospective cohort study

**DOI:** 10.3389/fendo.2024.1282015

**Published:** 2024-02-06

**Authors:** Jie Du, Wei Zhang, Jing Niu, Shuili Wang

**Affiliations:** ^1^ Department of Health Examination Center, Shaanxi Provincial People Hospital, Xi’an, China; ^2^ Department of Respiratory Medicine, Shaanxi Provincial People Hospital, Xi’an, China

**Keywords:** urea nitrogen, diabetes, adult, risk of disease, correlation

## Abstract

**Background:**

As one of the recognized indicators of kidney function, blood urea nitrogen (BUN) is a key marker of metabolic diseases and other diseases. Currently, data on the relationship of BUN levels with the risk of diabetes mellitus (DM) in Chinese adults are sparse. This study aimed to investigate the correlation between BUN levels and DM risk in Chinese adults.

**Data and methods:**

This study is a secondary analysis of a multicenter, retrospective cohort study with data from the Chinese health screening program in the DATADRYAD database. From 2010 to 2016, health screening was conducted on 211833 Chinese adults over the age of 20 in 32 locations and 11 cities in China, and there was no DM at baseline. Cox proportional hazards regression analysis assessed an independent correlation between baseline BUN levels and the risk of developing DM. The Generalized Sum Model (GAM) and smoothed curve fitting methods were used to explore the nonlinear relationship. In addition, subgroup analyses were performed to assess the consistency of correlations between different subgroups and further validate the reliability of the results.

**Results:**

After adjusting for potential confounding factors (age, sex, etc.), BUN levels were positively correlated with the occurrence of DM (HR=1.11, 95% CI (1.00~1.23)). BUN level had a nonlinear relationship with DM risk, and its inflection point was 4.2mmol/L. When BUN was greater than 4.2mmol/L, BUN was positively correlated with DM, and the risk of DM increased by 7% for every 1 mmol/L increase in BUN (P<0.05). Subgroup analysis showed that a more significant correlation between BUN levels and DM was observed in terms of sex, BMI, systolic blood pressure (SBP), diastolic blood pressure (DBP), total cholesterol (TC), triglycerides (TG), low-density lipoprotein (LDL), alaninetransaminase (ALT), aspartate transaminase (AST), creatinine (Cr) and smoking status (interaction P<0.05).

**Conclusion:**

High levels of BUN are associated with an increased risk of DM in Chinese adults, suggesting that active control of BUN levels may play an important role in reducing the risk of DM in Chinese adults.

## Introduction

Diabetes is a metabolic disorder characterized by elevated blood sugar levels, which can lead to various pathological changes (1). According to the data of the World Health Organization, the global population of diabetes is about 463 million, which is expected to exceed 500 million by 2030 (2). As a major global public health problem, DM is particularly prevalent in China (3). At present, the prevalence of diabetes in adults over 18 years old in China is as high as 11.2%, and the all-cause mortality rate of diabetic patients is 2~4 times that of non-diabetic patients, which has become one of the four major chronic diseases that cause death of residents, and it is crucial to formulate an effective DM prevention strategy (4, 5). In recent years, studies have found that elevated levels of circulating urea nitrogen (BUN) can directly damage pancreatic β cell function by increasing islet protein O-GlcNAcylation and impairing glycolytic processes, resulting in impaired glucose secretion, disruption of sugar homeostasis, occurrence of T2DM and decreased renal function. BUN may play an important role in the development of T2DM (6). Currently, few epidemiological studies have evaluated the association between BUN and the risk of DM in Chinese adults (7–9). Li et al. showed that BUN level was positively correlated with the risk of DM through a cohort study of 38578 adults in Beijing (4). Based on the small sample size and geographical differences of previous studies, this study based on a cohort of 211833 Chinese adults, and a secondary analysis based on the published available data was conducted to explore the potential relationship between BUN level and DM risk.

## Methods

### Participants selection and study grouping

A retrospective cohort study design was used for secondary analysis. The data came from the DATADRYAD database (www.DATADRYAD.org) provided by Chen et al (10). The study included Chinese adults over the age of 20 and covered 32 regions and 11 cities in China (Shanghai, Beijing, Nanjing, Suzhou, Shenzhen, Changzhou, Chengdu, Guangzhou, Hefei, Nantong and Wuhan) from 2010 to 2016.

### Data collection

A total of 211833 Chinese adults over the age of 20 were selected in the initial study who did not have diabetes at baseline, including 116123 men (54.82%) and 95,710 women (45.18%). The variables were as follows: age, sex, body mass index (BMI), diastolic blood pressure (DBP), systolic blood pressure (SBP), BUN, fasting blood glucose (FPG), triglycerides (TG), total cholesterol (TC), low-density lipoprotein cholesterol (LDL-C), high-density lipoprotein cholesterol (HDL-C), serum creatinine (Scr), alanine aminotransferase (ALT), aspartate aminotransferase (AST), year of follow-up, and diabetes screening at follow-up. All data is based on standardized conditions that follow uniform procedures.

### Diagnostic and exclusion criteria

DM is defined as fasting blood glucose≥7.00 mmol/L and/or self-reported DM during follow-up. Patients are tested at the time of diagnosis or at their last visit, whichever comes first; In this study, participants with missing BUN levels at baseline were excluded, and finally, 190282 participants were included in the secondary analysis.

The research programme is in line with the Declaration of Helsinki. In addition, the project was approved by various hospital institutional review boards, and access to patient data was anonymous and confidential due to the retrospective nature of the study. Since this study is a secondary analysis of a retrospective study, it has been approved by the Ethics Committee of Shaanxi Provincial People’s Hospital(2023-0632).

### Statistical methods

The mean of BUN was 4.658mmol/L, the median was 4.53mmol/L, the lowest was 0.1mmol/L, and the highest was 29.4mmol/L. Participants were grouped according to BUN quartiles (Q1:<0.692, Q2:0.692–1.093, Q3:11. 092–1.718, Q4:≥1.718). Continuous variables with normal distributions are represented by mean ± standard deviation (SD), skewed distributions by median, and frequency and percentage for categorical variables. Differences between different BUN groups were tested using χ2 (categorical variables) and one-way ANOVA (normal distribution) or Kruskal–Wallis H test (skewed distribution). To explore the association between baseline BUN levels and diabetes risk, univariate and multivariate Cox proportional hazards regression models were used after collinear screening, including unadjusted models (rough model: unadjusted covariate), minimally adjusted models (model I: adjusted age; Gender; BMI; SBP; DBP; CCR), and fully adjusted models (Model II: age-adjusted; Gender; BMI; SBP; DBP; CCR; FBG; TC; TG; HDL; LDL; ALT; AST; smoking status; Drinking status; family history). Effect sizes (HR) for 95% CI were calculated. A smoothed curve-fitting Cox proportional hazards regression model was used to show a nonlinear relationship between baseline BUN levels and DM risk. Finally, a log-likelihood ratio test is used to determine the most appropriate model. Multiple subgroups were analyzed using a hierarchical Cox proportional hazards regression model. Finally, a likelihood ratio test is used to determine whether there are interactions.

The study used R statistical software (https://www.r-project.org) and EmpowerStats (http://www.empowerstats.com, X&Y Solutions, Inc. Boston MA) to analyze the data. P<0.05 was statistically significant.

## Result

### Basic information characteristics when obtaining participant data

A total of 190282 Chinese adults over the age of 20 were included in this study (**Table 1**). Age, BMI, SBP, DBP, FBG, TC, TG, LDL-c, HDL-c, ALT, AST, and CCR increased significantly in the highest quartile compared to the lowest quartile (Q1:<0.692) (Q4:≥1.718). In addition, in the highest quartile, the proportion of men, current smokers, and current drinkers was higher (**Table 1**).

**Table 1:** Baseline characteristics of participants.

Variables	BUN quartile				F/χ^2^	*P-value*
	Q1(n=47545)	Q2(n=47509)	Q3(n=47579)	Q4(n=47649)		
Age(years)	38.97 ± 10.62	41.07 ± 11.87	42.85 ± 12.80	45.84 ± 14.20	0.174	<0.001
Gender, n(%)					-0.061	<0.001
Male	17319 (36.43%)	25007 (52.64%)	29122 (61.21%)	33153 (69.58%)		
Female	30226 (63.57%)	22502 (47.36%)	18457 (38.79%)	14496 (30.42%)		
BMI(kg/m^2^), Mean ± SD	22.53 ± 3.26	23.14 ± 3.34	23.47 ± 3.32	23.83 ± 3.30	0.010	<0.001
SBP(mmHg), Mean ± SD	116.13 ± 15.68	118.42 ± 16.17	119.93 ± 16.35	121.94 ± 16.92	0.020	<0.001
DBP(mmHg), Mean ± SD	72.61 ± 10.55	73.89 ± 10.74	74.64 ± 10.81	75.59 ± 10.96	0.032	<0.001
FBG (mmol/L), Mean ± SD	4.81 ± 0.59	4.88 ± 0.61	4.93 ± 0.61	5.03 ± 0.62	0.020	<0.001
TC(mmol/L), Mean ± SD	4.55 ± 0.86	4.68 ± 0.89	4.77 ± 0.91	4.84 ± 0.92	0.033	<0.001
TG(mmol/L), Mean ± SD	1.23 ± 0.89	1.33 ± 0.99	1.38 ± 1.05	1.42 ± 1.18	0.052	<0.001
HDL(mmol/L), Mean ± SD	1.39 ± 0.31	1.37 ± 0.31	1.36 ± 0.31	1.37 ± 0.31	-0.008	<0.001
LDL(mmol/L), Mean ± SD	2.64 ± 0.65	2.75 ± 0.67	2.81 ± 0.69	2.86 ± 0.69	0.033	<0.001
ALT(U/L), Mean ± SD	21.33 ± 23.40	23.87 ± 22.63	24.99 ± 21.30	25.80 ± 20.73	0.025	<0.001
AST(U/L), Mean ± SD	23.15 ± 14.73	23.94 ± 12.55	24.36 ± 11.71	24.80 ± 9.91	0.002	<0.001
Cr(umol/L), Mean ± SD	63.31 ± 13.48	68.76 ± 14.36	72.02 ± 14.69	75.94 ± 17.20	0.242	<0.001
DM					0.039	<0.001
No	46906 (98.66%)	46696 (98.29%)	46581 (97.90%)	46303 (97.18%)		
Yes	639 (1.34%)	813 (1.71%)	998 (2.10%)	1346 (2.82%)		
Smoking Status					0.108	<0.001
often	1886 (14.02%)	2640 (19.22%)	3006 (21.96%)	3371 (24.96%)		
once	432 (3.21%)	561 (4.08%)	645 (4.71%)	710 (5.26%)		
never	11132 (82.77%)	10533 (76.69%)	10037 (73.33%)	9427 (69.79%)		
Drinking Status					0.090	<0.001
often	190 (1.41%)	281 (2.05%)	311 (2.27%)	435 (3.22%)		
once	1470 (10.93%)	2049 (14.92%)	2231 (16.30%)	2550 (18.88%)		
never	11790 (87.66%)	11404 (83.03%)	11146 (81.43%)	10523 (77.90%)		
Family History of Diabetes					-0.015	<0.001
No	46415 (97.62%)	46452 (97.78%)	46652 (98.05%)	46777 (98.17%)		
Yes	1130 (2.38%)	1057 (2.22%)	927 (1.95%)	872 (1.83%)		

BMI, body mass index; SBP, systolic blood pressure; DBP, diastolic blood pressure; TC, total cholesterol; TG, triglycerides; HDL, high-density lipoprotein; LDL, low-density lipoprotein; ALT, alaninetransaminase; AST, aspartate transaminase; Cr, creatinine; DM, diabetes mellitus. BUN, blood urea nitrogen.

### Univariate Cox proportional hazard regression analysis

Age, BMI, SBP, DBP, FBG, TC, TG, LDL, AST, BUN, CCR, and family history were positively associated with risk in patients with DM, but gender, HDL, smoking status, and alcohol consumption status were negatively associated with risk in patients with DM (P<0.05; **Table 2**).

**Table 2:** Results of univariate Cox proportional hazard regression analysis of BUN level and DM risk in Chinese adults.

Variables	DM risk	
	*HR* (95% CI)	*P-value*
Age	1.07 (1.06, 1.07)	<0.0001
Gender		
Male	1.0	
Female	0.49 (0.45, 0.52)	<0.0001
BMI	1.24 (1.23, 1.25)	<0.0001
SBP	1.04 (1.04, 1.04)	<0.0001
DBP	1.05 (1.04, 1.05)	<0.0001
FBG	10.45 (10.00, 10.91)	<0.0001
TC	1.43 (1.39, 1.47)	<0.0001
TG	1.26 (1.25, 1.28)	<0.0001
HDL	0.60 (0.53, 0.68)	<0.0001
LDL	1.35 (1.28, 1.42)	<0.0001
ALT	1.00 (1.00, 1.00)	<0.0001
AST	1.01 (1.01, 1.01)	<0.0001
BUN	1.24 (1.21, 1.26)	<0.0001
BUN quartile		
Q1	1.0	
Q2	1.25 (1.13, 1.38)	<0.0001
Q3	1.54 (1.40, 1.71)	<0.0001
Q4	2.06 (1.87, 2.26)	<0.0001
Cr	1.01 (1.01, 1.01)	<0.0001
Smoking Status		
often	1.0	
once	0.79 (0.62, 1.01)	0.0565
never	0.44 (0.39, 0.49)	<0.0001
Drinking Status		
often	1	
once	0.47 (0.34, 0.65)	<0.0001
never	0.47 (0.35, 0.62)	<0.0001
Family History of Diabetes		
No	1.0	
Yes	1.73 (1.48, 2.01)	<0.0001

BMI, body mass index; SBP, systolic blood pressure; DBP, diastolic blood pressure; TC, total cholesterol; TG, triglycerides; HDL, high-density lipoprotein; LDL, low-density lipoprotein; ALT, alaninetransaminase; AST, aspartate transaminase; Cr, creatinine; DM, diabetes mellitus. BUN, blood urea nitrogen.

### Relationship between baseline BUN level and the risk of DM

Three models were constructed using the Cox proportional hazards regression model. In the unadjusted model (rough model), the risk of DM increased by 25% for each increase in baseline BUN level by 1 mmol/L (HR = 1.25, 95% CI: 1.22~1.28, P<0.005). In Model I (adjusted for age, sex, MI, SBP, DBP and CCR), for each 1 mmol/L increase in baseline BUN level, the risk of DM increased by 10% (HR = 1.10, 95% CI: 1.04~1.16), P<0.005). In model II (adjusted for age, sex, BMI, SBP, DBP, CCR, FBG, TC, TG, HDL, LDL, ALT, AST, SMOKING STATUS, DRINKING STATUS, AND FAMILY HISTORY), THE RISK OF DM INCREASED BY 11% FOR EACH INCREASE IN BASELINE BUN LEVEL (HR=1.11, 95%CI: 1.00~1.23, P<0.005). The distribution of confidence intervals suggests that the relationship between baseline BUN levels and DM risk in Chinese adults obtained by this model is reliable. To confirm the robustness of the results, sensitivity analyses were performed on baseline BUN levels by categorical variables (quartile groupings). The results showed that BUN levels were positively correlated with the risk of diabetes (**Table 3**).

**Table 3:** Cox proportional hazard regression model of BUN level and DM risk in Chinese adults.

Variables	Non-adjusted		Adjust I		Adjust II	
	*HR* (95% CI)	*P-value*	*HR* (95% CI)	*P-value*	*HR* (95% CI)	*P-value*
BUN	1.25 (1.22, 1.28)	<0.0001	1.10 (1.04, 1.16)	0.0008	1.11 (1.00, 1.23)	0.0423
BUN quartile						
Q1	1.0		1.0		1.0	
Q2	1.28 (1.15, 1.42)	<0.0001	0.91 (0.73, 1.12)	0.3632	0.89 (0.58, 1.38)	0.6118
Q3	1.57 (1.42, 1.74)	<0.0001	1.08 (0.89, 1.33)	0.4299	0.98 (0.66, 1.47)	0.9307
Q4	2.13 (1.94, 2.35)	<0.0001	1.19 (0.98, 1.45)	0.0827	1.14 (0.77, 1.67)	0.5148
*P* for trend	1.29 (1.25, 1.32)	<0.0001	1.08 (1.02, 1.15)	0.0103	1.06 (0.94, 1.20)	0.3253

### Nonlinear relationship analysis

**Figure 1:**
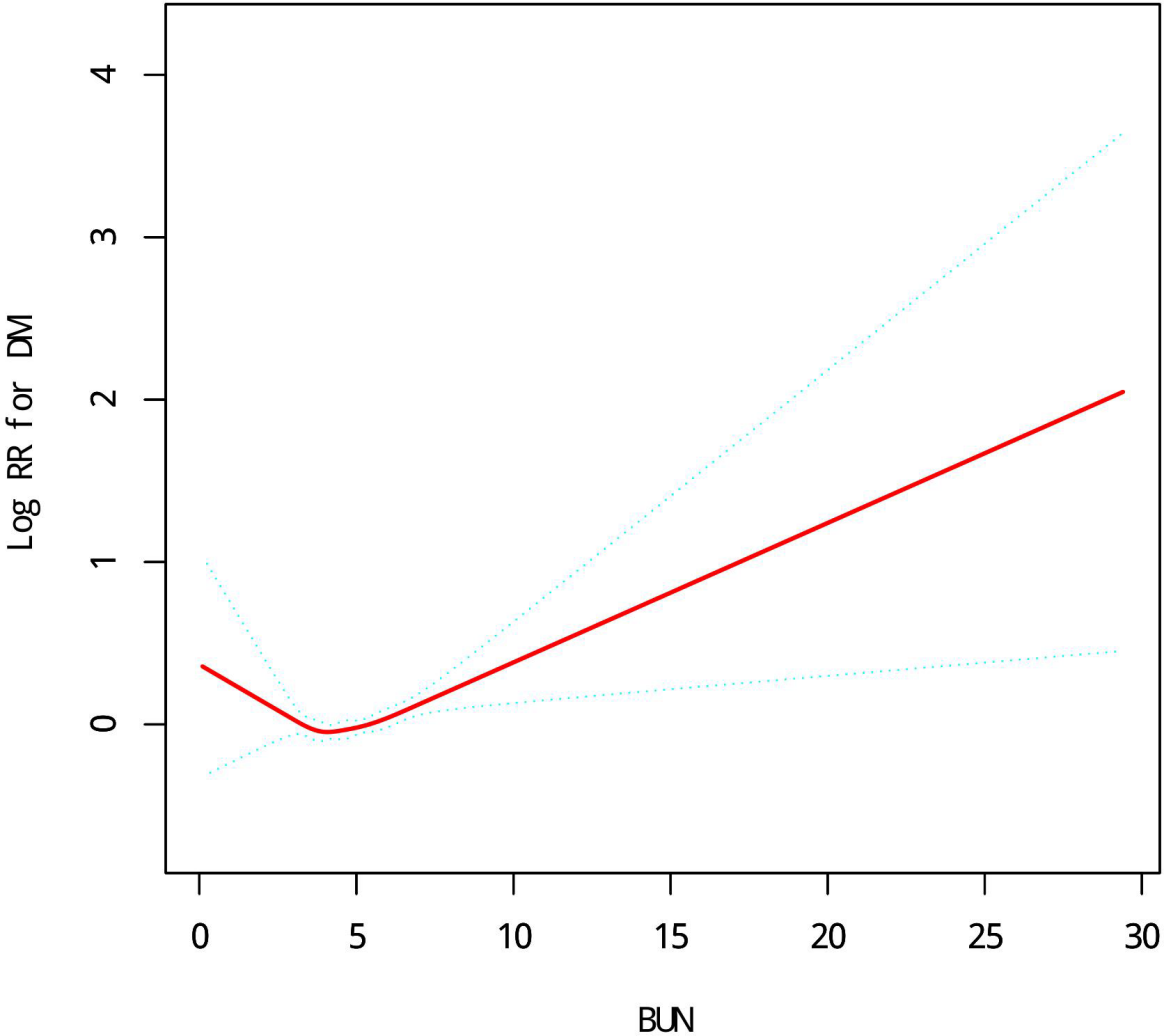
A non-linear relationship of BUN levels with DM risk in Chinese adults.

Through polynomial Cox proportional hazards regression model analysis, after adjusting for age, sex, BMI, SBP, DBP, TC and CCR, the nonlinear relationship between baseline BUN level and DM risk was found (**Figure 1**). Through the analysis of the two-stage Cox proportional hazards regression model, it is found that the inflection point is 4.2mmol/L, and the effect size and confidence interval on the right side of the inflection point are 1.07(1.04~1.11, P<0.05). However, on the left side of the inflection point, no association between BUN levels and DM risk was observed (P=0.075) (**Table 4**).

**Table 4:** The results of two-piecewise linear regression model.

Models	HR (95% CI)	P-value
Model I		
One line slope	1.04 (1.01, 1.07)	0.0030
Model II		
Inflection point	4.2	
<4.2	0.91 (0.83, 1.01)	0.0750
>4.2	1.07 (1.04, 1.11)	<0.0001
LRT test		0.008

Model I, linear analysis; Model II, nonlinear analysis. LRT test, logarithmic likelihood ratio test (p value < 0.05 indicates that Model II is significantly different from Model I, which indicates a nonlinear relationship).

### Subgroup analysis

**Figure 2:**
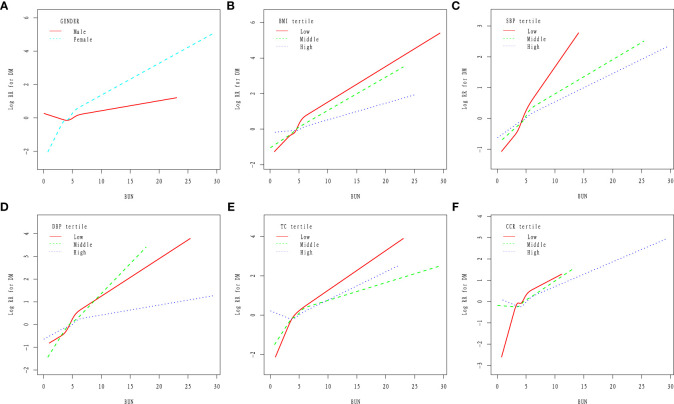
**(A-F)** Effect of BUN levels on DM risk in Chinese adults by subgroups of gender, BMI, SBP, DBP, TC and CR.

To further analyze the impact of other risk factors associated with baseline BUN levels and risk of DM, the following stratified variables were analyzed in this study: age, gender, BMI, etc. Stronger correlation interactions were found in gender, BMI, SBP, DBP, TC, TG, LDL, ALT, AST, CCR, SMOKING STATUS, AND DRINKING STATUS (P<0.05). (**Figures 2A–F**).

## Discussion

This study found that an increase in baseline BUN levels in Chinese adults was associated with a significant increased risk of DM. In addition, a threshold effect of curve fitting was also found, and when the baseline BUN level was equal to 4.2mmol/L, the effect size and confidence interval on the right side of the inflection point were 1.07(1.04~1.11 P<0.05). However, on the left side of the inflection point, no association between BUN levels and DM risk was observed (P=0.075). The robustness of the results was further demonstrated by subgroup analysis, and stronger correlation interactions were found in sex, BMI, SBP, DBP, TC, TG, LDL, ALT, AST, CCR, and smoking status (P<0.05).

BUN is a protein metabolism of the end produced by the liver and excreted by the kidneys, and it is used as a biomarker for the periodic assessment of kidney function (11, 12). It has long been thought that its toxicity is negligible. However, the idea that urea is just an innocent bystander has recently been challenged by several new ideas (13–15). Du et al. showed that BUN levels at admission were closely correlated with the length of hospital stay of AECOPD patients, and when BUN levels were less than 40 mg/dl, length of stay was positively correlated with BUN levels (16). Canalin et al. investigated the NHANES database of adults in the United States and found a clear correlation between BUN levels and cardiovascular disease prevalence and all-cause mortality (17). In addition, protein intake, corticosteroid levels, gastrointestinal bleeding, and dehydration are several factors that alter BUN levels. BUN can indicate a link between nutritional status, protein metabolism, and renal function, and is a key marker of metabolic and other diseases (18). BUN helps reduce insulin sensitivity and plays an important role in the urine concentration process and water preservation in mammalian kidneys (19, 20). Results from numerous cross-sectional and longitudinal population studies suggest that blood BUN levels are associated with an increased risk of diabetes (7–9), which is consistent with higher urea levels in the diabetes group in this study population.

BUN is recognized as one of the indicators of renal function, and previous experimental studies have shown that high concentrations of BUN can increase reactive oxygen species (ROS) in the medulla (mIMCD3) cells of mice, resulting in insulin resistance in fat cells, which is closely related to the development of DM (21, 22). Elevated urea nitrogen levels *in vivo* may induce insulin resistance, inhibit insulin secretion, reduce glycolytic flux, and attenuate insulin signaling (23, 24). In addition, studies have shown that some metabolites of BUN, including arginine, citrulline, and ornithine, are associated with the risk of T2DM in Chinese adults, and that the arginine/ornithine, arginine/citrulline, and citrulline/ornithine ratios are significantly increased in patients with TDM2 compared with controls (25). The correlation between elevated BUN concentrations and disease may be related to the following mechanisms: renal dysfunction to some extent represents acute and predominantly hemodynamic renal impairment. In the absence of urea boosters, such as gastrointestinal bleeding, corticosteroid therapy, or a high-protein diet, an increase in BUN levels is usually due to a decrease in glomerular filtration rate. Higher BUN levels at baseline may indicate renal disease and have been associated with coronary artery disease (26). On the other hand, BUN not only reflects kidney function, but is also associated with neurohormonal activation (27). The increase in BUN reflects the cumulative effect of hemodynamic and neurohormonal changes, which leads to hypoperfusion of the kidneys. A large cohort of 1337452 U.S. veterans, with a mean follow-up of 4.93 years, found that higher levels of BUN were associated with an increased risk of developing DM in non-DM populations (28), and higher BUN levels were associated with an increased risk of insulin use in patients with diabetes (29); Another cross-sectional study of 3227 women and 610 patients with T2DM also showed a positive association with T2DM. Pei et al. conducted a prospective, multicenter cohort study of 13,448 pregnant women and showed that higher BUN concentrations in the first trimester of pregnancy were associated with an increased risk of DM during pregnancy (7). Our study shows a positive nonlinear relationship between baseline BUN levels and DM risk in Chinese adults. This result is expected to provide a reference for clinicians to actively control abnormal BUN levels in patients.

### Strength of the study

This study has several advantages: (i) The total sample size is relatively large. (ii) To our knowledge, this is the first time that Chinese adults have been used as a study population to explore the relationship between baseline BUN levels and DM risk. And (iii) significant improvements in studies targeting nonlinearity compared to previous studies.

### Limitations of the study

There are also some limitations to this study: this is a retrospective study and no data can be obtained before diabetes diagnosis, so conclusions cannot be drawn about the causal relationship between risk factors and blood glucose abnormalities. BUN is a single measurement that may be influenced by other factors such as protein intake and hydration status. However, such related variables were not collected in the original data. Perhaps because the original data included a relatively large national representative sample of Chinese adults, some confounding factors were not collected. Although we adjusted for a wide range of confounding factors, residual confounding factors due to measurement errors in confounding factor assessment, as well as unmeasured factors such as physical activity and dietary factors, cannot be excluded. Collecting as many variables as possible for further exploration in future clinical and epidemiological studies may be considered.

## Conclusion

In conclusion, when the baseline BUN level was equal to 4.2mmol/L, the effect size and confidence interval on the right side of the inflection point were 1.07(1.04~1.11 P<0.05). These findings indicate a significant correlation between BUN and DM risk, and although we have provided some clinical clues, further prospective studies are needed to provide more evidence.

## Data availability statement

Publicly available datasets were analyzed in this study. This data can be found here: https://datadryad.org/stash/dataset/doi:10.5061/dryad.ft8750v.

## Ethics statement

The studies involving humans were approved by the Ethics Committee of Shaanxi Provincial People’s Hospital. The studies were conducted in accordance with the local legislation and institutional requirements. Written informed consent for participation was not required from the participants or the participants’ legal guardians/next of kin in accordance with the national legislation and institutional requirements. Written informed consent was not obtained from the individual(s) for the publication of any potentially identifiable images or data included in this article because this study is a secondary analysis of a retrospective study.

## Author contributions

JD: Conceptualization, Writing – original draft. WZ: Data curation, Writing – review & editing. JN: Methodology, Formal Analysis, Writing – original draft. SW: Project administration, Supervision, Writing – review & editing.
